# Integrating mental health into primary care in Nigeria: report of a demonstration project using the mental health gap action programme intervention guide

**DOI:** 10.1186/s12913-015-0911-3

**Published:** 2015-06-21

**Authors:** Oye Gureje, Jibril Abdulmalik, Lola Kola, Emmanuel Musa, Mohammad Taghi Yasamy, Kazeem Adebayo

**Affiliations:** WHO Collaborating Centre for Research and Training in Mental Health and Neuroscience, Department of Psychiatry, University of Ibadan, Ibadan, Nigeria; Department of Psychiatry, University of Ibadan, Nigeria, Ibadan, Nigeria; World Health Organization, Nigeria Country Office, Osogbo, Nigeria; Nigeria Country Office, World Health Organization, Abuja, Nigeria; Department of Mental Health and Substance Abuse, World Health Organization, Geneva, Switzerland; Department of Psychiatry, Ladoke Akintola University of Technology, Ogbomoso, Nigeria

**Keywords:** Integration, Mental health, Primary care, Nigeria, MHGAP-IG

## Abstract

**Background:**

The World Mental Health Surveys conducted by the World Health Organization (WHO) have shown that huge treatment gaps for severe mental disorders exist in both developed and developing countries. This gap is greatest in low and middle income countries (LMICs).

Efforts to scale up mental health services in LMICs have to contend with the paucity of mental health professionals and health facilities providing specialist services for mental, neurological and substance use (MNS) disorders. A pragmatic solution is to improve access to care through the facilities that exist closest to the community, via a task-shifting strategy. This study describes a pilot implementation program to integrate mental health services into primary health care in Nigeria.

**Methods:**

The program was implemented over 18 months in 8 selected local government areas (LGAs) in Osun state of Nigeria, using the WHO Mental Health Gap Action Programme Intervention Guide (mhGAP-IG), which had been contextualized for the local setting.

A well supervised cascade training model was utilized, with Master Trainers providing training for the Facilitators, who in turn conducted several rounds of training for front-line primary health care workers. The first set of trainings by the Facilitators was supervised and mentored by the Master Trainers and refresher trainings were provided after 9 months.

**Results:**

A total of 198 primary care workers, from 68 primary care clinics, drawn from 8 LGAs with a combined population of 966,714 were trained in the detection and management of four MNS conditions: moderate to severe major depression, psychosis, epilepsy, and alcohol use disorders, using the mhGAP-IG. Following training, there was a marked improvement in the knowledge and skills of the health workers and there was also a significant increase in the numbers of persons identified and treated for MNS disorders, and in the number of referrals. Even though substantial retention of gained knowledge was observed nine months after the initial training, some level of decay had occurred supporting the need for a refresher training.

**Conclusion:**

It is feasible to scale up mental health services in primary care settings in Nigeria, using the mhGAP-IG and a well-supervised cascade-training model. This format of training is pragmatic, cost-effective and holds promise, especially in settings where there are few specialists.

**Electronic supplementary material:**

The online version of this article (doi:10.1186/s12913-015-0911-3) contains supplementary material, which is available to authorized users.

## Background

The gap between need and available services for mental health care is big in most countries, but especially so in low- and middle-income countries (LMIC). The World Mental Health Surveys conducted by the World Health Organization (WHO) suggests that the treatment gap for severe mental disorders in LMIC can be as large as 75 % [1]. About 80 % of persons with epilepsy are from LMIC, out of whom about 6 in 10 do not receive any treatment [2]. These conditions are associated with considerable disability for the patients as well as a high burden for their families [3]. In Sub Saharan Africa (SSA), the added social alienation that results from the stigmatization of mental, neurological and substance use (MNS) disorders exposes the sufferers to abuse and economic impoverishment [4].

It is generally agreed that the best way to scale up service for persons with identified need for mental health care is to integrate the care into primary care [5, 6]. By conceptualization, primary care is expected to offer first contact, and in addition, to providing comprehensive, continuous, and coordinated service for persons with health conditions, it should also display the capacity for prompt referrals to a higher level of care. However, this expectation is hardly achieved for MNS conditions within the primary care service in the majority of LMIC. The reasons include inadequate training of primary care providers, leading to poor recognition and inappropriate treatment of mental health conditions, absence of support and supervision for their work, uncoordinated referral pathway through the various tiers of the health service, and policy neglect that commonly manifests in the form of poor funding, irregular supply of medications for MNS conditions [7–10] and weak health systems [11].

The call for the integration of mental health into primary care is not without criticism however. It has rightly been argued that integration should not be presented as a magic bullet that will solve all the ills of the treatment gap for MNS disorders in LMICs. The view has also been expressed that integration may impose western medical treatment models, pay scant attention to other sectors of health care (such as the district or secondary tier levels) and result in a failure to address and strengthen community rehabilitation [12, 13]. The experience from other SSA countries have also raised concerns about challenges of integration such as poor policy implementation, insufficient numbers of mental health professionals to drive and support the process, poor community engagement and mobilization, and the non-availability of medications [10, 14].

### Local context of mental health services

In Nigeria, as in most sub-Saharan African countries, there is a gross inadequacy of mental health specialist services. For example, Nigeria has about 200 psychiatrists to its population of 170 million. Most of these specialists work in a few urban centres leaving the vast majority of the country’s communities with no specialist service. A recent large-scale epidemiological study in several countries (including Nigeria) have shown that only about 20 % of persons with common but serious mental disorders, such as depression with suicidal risk, had received any treatment in the prior 12 months. Among those who did, only 10 % received minimally adequate treatment [1].

The vast majority of those who seek care often do so from traditional and spiritual healers, and when that fails, they subsequently present at non-specialist settings, especially at those facilities closest to their communities: the primary care facilities. In recognition of the strategic importance of primary health care facilities to the improvement of access to mental health services, the government of Nigeria has committed itself to the integration of mental health service into primary care [15].

Primary health care service in Nigeria, similar to what obtains in most sub-Saharan African countries, is manned by various cadres of non-physician community health workers. These workers have commonly received 2–3 years of post-high school training in community health care services. However, this training has not adequately provided them with the skills required to recognize and treat MNS conditions commonly encountered in primary care, such as psychosis, severe depression and epilepsy. There is now evidence from previous studies within the Nigerian setting, that such workers can be trained to acquire such skills and to deliver both psychological and pharmacological treatments [16, 17].

An added barrier to the provision of adequate mental health service in the Nigerian setting is the lack of coordination of mental health services across the tertiary, secondary and primary tiers of the health system. The most important aspect of this poor coordination is that movement of patients from one level to the other is not properly organized to ensure that needs determine the extent of service provided. For example, a national survey showed that a disproportionate number of persons with mild mental health conditions received specialist care while those with more severe conditions continue to receive inadequate care at the primary care level [1]. Thus, the system is characterized by inequity and inefficiency [18].

## Methods

### The project overview

The World Health Organization’s (WHO) Mental Health Gap Action Programme (mhGAP) is designed to provide a roadmap for governments, especially those of LMIC, to implement comprehensive mental health service reform that will address the challenges that hinder effective provision of care to those in need [19]. The programme includes steps that will enhance patient care at the level of service delivery to those that provide policy framework for sustained mental health service improvement, including supply of medication. A major component of the programme is the mhGAP-Intervention Guide (mhGAP-IG) which is designed to assist non-specialists to recognize and offer evidence-based treatment to persons with a range of nine priority mental, neurological and substance use disorders [20].

In order for the mhGAP to achieve its programmatic goal, there are a number of important steps that need to be taken. Firstly, the WHO recommends that the mhGAP-IG be contextualized and adapted for the setting in which it is to be used. Contextualization is particularly important in settings, like those obtainable in much of Sub-Saharan Africa, where most primary care providers are not only non-specialists, but are also mostly non-physician community health workers. This step has already been successfully completed in Nigeria [21]. Secondly, given that a crucial step in efforts to scale up mental health service is the training of primary care providers, users of mhGAP-IG are required to be trained in its use. Again, effective and well-conducted training is particularly necessary where the end users of the guide are non-physician community health workers. While the WHO has prepared and made available several modules of training packages, there remains the need to find trainers to offer the training. In LMIC, where specialists are few and often engrossed in the daily routine of providing care for the teeming persons in need, such specialists are hardly available to provide training for the large number of end users (primary care workers) who need to be so trained in order for effective impact to be made on service delivery. This project has addressed this challenge by conducting a series of well supervised cascade training for primary care workers with the use of the contextualized mhGAP-IG. Ethical approval to engage the facilities, conduct the training and assess its impact was obtained from the Ethics and Research Committee of the Osun State Ministry of Health. Written informed consent was also obtained from the head of each facility included in the study followed by verbal consent from the participating providers. The primary care workers who participated in the trainings provided only verbal consent because this was a health-system project with only facility-level data required. The numbers consenting to participate were documented in writing, for each of the facilities involved in the implementation project. No personal information or individual patient-level data were collected. This consent procedure was approved by the Ethics and Research Committee of the Osun State Ministry of Health.

### Project design and implementation

The project aim was to demonstrate the feasibility and acceptability of using the adapted mhGAP-IG to scale up services for MNS disorders through a program of training of primary care workers followed by active monitoring and supervision of these workers by physicians and specialists. The program was implemented in 8 selected local government areas in Osun, one of the 36 states of Nigeria. It was designed as a pilot demonstration project in conjunction with the Federal Ministry of Health (FMoH) and the Osun State Ministry of Health (MoH) to evaluate the feasibility of this model for scaling up mental health services across the country. The goal was to determine the organizational and logistic issues that might impact the delivery of evidence-based intervention for selected MNS disorders, using the mhGAP-IG, by frontline providers in their routine practice.

#### a). *Planning*

The project commenced with three planning workshops. The workshop participants were key players in mental health service delivery in the country as well as primary care providers and policy makers. The aims of the workshops included: 1). the detailed planning for the proposed project 2) the development of course materials and design of monitoring and evaluation activities and 3) the design of the public and policy engagement activities that would ensure project buy-in and sustainability.

#### b). *Implementation*

The first training workshop was a direct training of the end users, the primary care providers, by the Master Trainers who were mental health specialists. This first workshop was designed to pilot test the instructional methods to be used, including role plays, to train users in the application of the mhGAP-IG. The series of training workshops that followed utilized a cascade format. The goal was to deliver acceptable level of skill acquisition and fidelity while maximizing the use of the limited available specialist resources. The basic structure consisted of: 1) the training of a core set of Trainers (henceforth referred to as Facilitators for ease of communication) by a group of Master Trainers. The Facilitators were non-specialist physicians and senior nurses while the Master Trainers were psychiatrists. The training of the Facilitators covered all the 9 modules of the mhGAP-IG as well as techniques of teaching, including how to organize and conduct role plays to enhance clinical skills acquisition. 2) The Master Trainers contextualized and adapted the training materials to make them appropriate for use by the Facilitators. The materials were then provided to the Facilitators for their subsequent use, thus relieving them of what would have been the additional burden of having to prepare these materials themselves; 3) At least one Master Trainer sat in and observed the initial training sessions conducted by the Facilitators. The presence of a Master Trainer at the initial trainings was aimed at providing direct support, and to help with addressing the more difficult questions that might arise from the trainee participants; 4) At the end of the initial trainings, a de-briefing exercise was held with the Facilitators during which observations made during the training sessions were discussed and further clarifications provided.

A refresher workshop was held mid-way into project implementation. It was specifically structured to relate the reinforcement of knowledge and skills, to the clinical experience already acquired by the providers during their routine practice. Specifically, discussions were based on what had been observed in the clinical settings and the challenges that providers had faced in conducting patient assessment and offering treatment. At the beginning of the 2-day workshop, a pre-training test was conducted along the lines of the original pre- and post-tests conducted before the initial training several months earlier (see Additional file 1).

#### c). *Monitoring and evaluation*

Monitoring and supervision activities were designed to support the trained staff in the effective utilization of their acquired skills to deliver mental health care services. It consisted of regular clinic visits by the project staff to conduct live clinical observations, review patients’ clinical notes, and to hold debriefing sessions with the clinical staff. The sessions were aimed at addressing emerging clinical challenges and sharing of experience among the providers. Facilitators served as the main supervisors since they were already within the primary care system and could therefore provide sustainable input. They were supported by the mental health specialists who made less frequent but scheduled and targeted visits during which they held debriefing sessions with the supervisors and frontline providers, attending to issues arising from real clinical challenges that might have been encountered. These monitoring activities were structured and clearly defined to ensure the quality of service provided was consistently high and thereby enhance the prospects of effective impact. Monitoring and supervision was a continuous activity throughout the duration of the project. The monitoring activities consisted of: 1) reviewing clinical notes to ensure that proper documentation is made of every clinical encounter; 2) non-intrusive observation by a supervisor of the process of clinical assessment of patients by the health providers using the mhGAP-IG; 3) debriefing meetings with the clinical staff to discuss what has been observed or noted in (1) and (2). In general, monitoring exercise sought to re-inforce skills acquired during training and to promote fidelity in the use of the mhGAP guidelines.

Evaluations were conducted using the WHO mhGAP monitoring and evaluation toolkit, which was contextualized for the Nigerian setting. These evaluations, conducted midway and at the end of the project, followed a well-structured format and consisted of detailed information about fidelity, clinical documentation, patient flow and referral process.

## Results

A total of 198 primary care workers, from 68 primary care clinics, drawn from 8 out of the 30 Local Government Areas (LGAs) of Osun State were trained in the detection and management of four MNS conditions: moderate to severe major depression, psychosis, epilepsy, and alcohol use disorders, using the mhGAP-IG. The selection of the disorders was made through a process of consultation with primary care providers in the study setting, using the criteria of their prevalence and presentation to the primary care service, severity, associated social stigma, and availability of effective and affordable interventions.

**Fig. 1:**
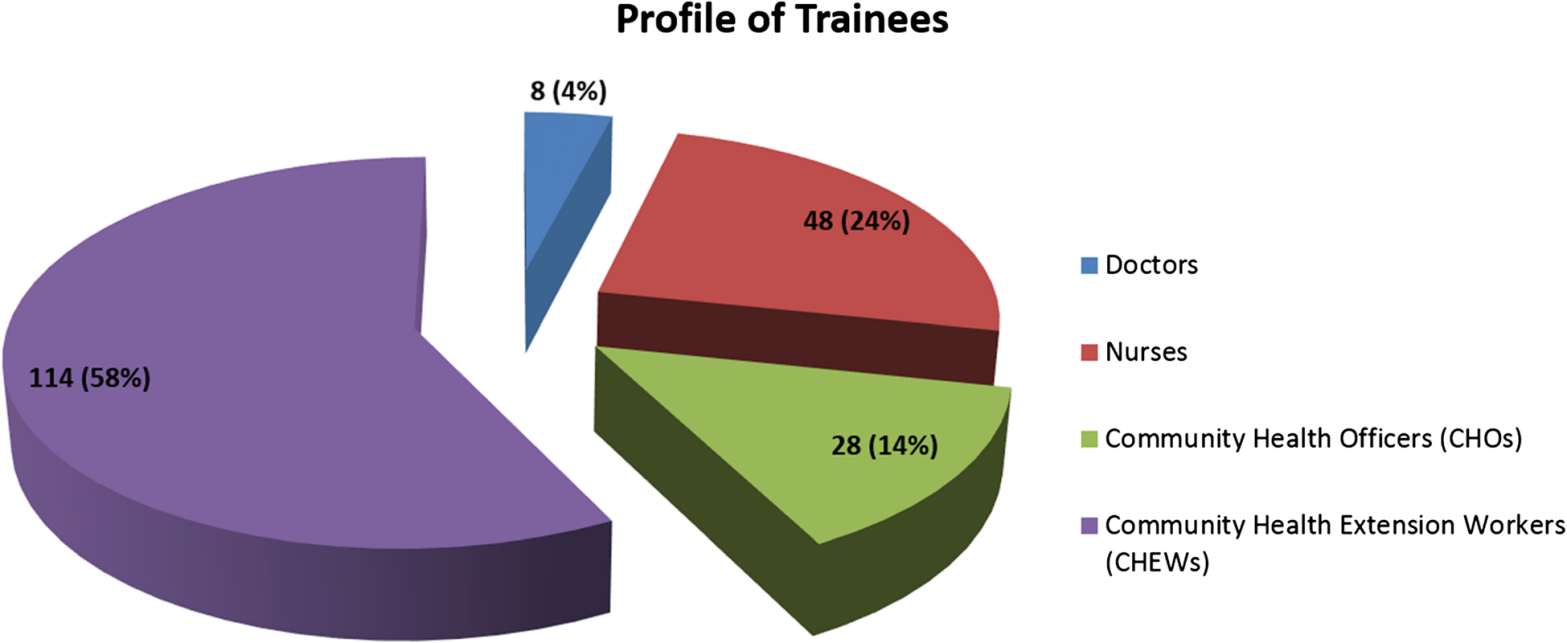
Professional profile of trainee primary care staff

The selected LGAs comprised of 4 rural, 2 semi-urban and 2 urban locations with a combined population of 966,714, which accounts for a third of the state’s population. The profile of the professional cadres of trainees is depicted in Fig. 1.

As shown in Table 1, the following observations were made:

1. Trainees showed significant improvement in acquired knowledge as demonstrated by pre- and post-tests, using a modified standardized assessment designed by the WHO for trainings on the MHGAP.
2. There was adequate retention of acquired skills as shown by a repeat test conducted 9 months after initial training.
3. There was nevertheless, evidence of some decay in acquired skills over 9 months as reflected in lower mean scores at 9 months, as compared to the initial post training scores at baseline.

**Table 1:** Test scores comparisons; pre and post training and after 9 months

Comparisons	Mean score differences	P	CI
Initial pre- and post- training tests	−4.90	<0.001	−5.88 to −3.92
Initial pre-training scores versus scores 9 months later	−2.92	<0.001	−3.96 to −1.88
Initial post-training scores versus scores 9 months later	1.98	<0.001	1.95 to 3.01

As shown in Table 2, compared to the situation before program implementation when there was no MNS detection and treatment activity in these clinics, the trained staff successfully identified and treated 96 clients. Furthermore, there was a substantial increase in the MNS referrals made to the other levels of care, including to psychiatrists, from a baseline of zero to a total of 45 referrals. These referrals were also efficiently coordinated along established channels.

**Table 2:** Summary of individuals receiving care for MNS disorders in participating clinics

	No of patients with MNS treated in Primary Care Clinics	No of patients with MNS referred to PHC doctor	No of patients referred to psychiatrists	Total numbers presenting for follow-up clinics
Baseline assessment in 2011	DEP - 0	DEP – 0	DEP – 0	-
	PSY - 0	PSY - 0	PSY - 0	
	EPI - 0	EPI - 0	EPI - 0	
	DEV - 0	DEV - 0	DEV - 0	
	ALC - 0	ALC - 0	ALC - 0	
	TOTAL – 0	TOTAL - 0	TOTAL – 0	
Repeat assessment in November 2013	DEP - 45	DEP – 8	DEP - 2	
	PSY- 13	PSY- 2	PSY- 4	
	EPI- 14	EPI- 8	EPI- 2	
	DEV- 4	DEV- 3	DEV- 3	
	ALC- 5	ALC- 2	ALC- 0	
	TOTAL = 96	TOTAL = 31	TOTAL = 14	51

MNS is mental, neurological and substance use disorders

DEP is depression

PSY is psychosis

EPI is epilepsy

DEV is developmental disorders

ALC is alcohol use disorders

In regard to fidelity, cumulatively over the period of evaluation, about 65 % of observed or assessed clinical encounters were rated to be in substantial compliance with the mhGAP-IG in the assessment and treatment of patients.

Other indices of impact:

1. Supply of psychotropic medications in the clinics as well as in the adjoining private pharmacies improved substantially, driven by increased demand.
2. Policy attention to mental health issues at the level of the state government improved remarkably as shown by (a) the employment of the first psychiatrist in the state; and (b) the landmark adoption of a national implementation plan for scaling up mental health service using the mhGAP-IG at the 56th National Council of Health meeting in August, 2013. The Council consists of all the Health Commissioners across the country’s 36 states and is chaired by the Federal Minister of Health.

## Discussion

We report experience gained in the course of implementing a program of work to demonstrate the feasibility of using the mhGAP-IG to scale up mental health service in Nigeria. The mhGAP-IG is a new tool developed by the WHO to facilitate the delivery of evidence-based intervention for a set of priority MNS conditions by non-specialists, especially those working in resource-constrained settings. To the best of our knowledge, this report represents the first attempt to document the utility of using the mhGAP-IG to deliver intervention in routine practice in primary care.

The project was implemented in Osun state of Nigeria. The state was selected for the demonstration project in view of its peculiarities in the context of mental health service in Nigeria. Even though it has only 8 psychiatrists to its population of over 3 million people, it is the only state in Nigeria with specialist mental health facilities in three of its cities. This makes it ideally suited for testing the implementation of a coordinated mental health service in which referral pathways through the three tiers of service (primary, secondary and tertiary) can be evaluated.

This project has demonstrated the feasibility of using a specially designed cascade training to build the skills of primary care workers in the use of mhGAP-IG. The approach ensured that there was substantial retention of the quality of training through the cascade and in the knowledge and skills acquired by attending trainees. Compared to direct training, cascade training runs the risk of quality decay. However, direct training of end users requires a large and regular supply of specialists to conduct the training, in several batches. This is neither feasible nor sustainable in low and middle income countries such as in Nigeria, where there are very few specialists who are often over-burdened with clinical and administrative responsibilities. This study demonstrates that cascade training, modified as described in this demonstration project, may be a feasible option of training non-physician primary care providers to implement evidence-based guidelines for patient care. In places where a few specialists are available, they can only implement a limited number of training workshops because of other demands on their time. Utilizing such limited workshop sessions to build a middle-level cadre of trainers not only ensures that training and re-training can be done, but also builds a layer of secondary-level providers who can deliver monitoring and evaluation activities and also provide clinical expertise along the referral pathway. This approach provides a sustainable, cheap and yet effective approach to imparting the skills necessary to implement the mhGAP guidelines for patient care. Even in settings where specialists are not available, what is required is a one-off effort to get such Master Trainers, possibly invited from external sources, to build the required cadre of local trainers. This approach holds promise and it enhances the prospects of mhGAP-IG to be potentially relevant to the local settings in many low- and middle-income countries.

An important monitoring aspect of the project is the conduct of refresher workshops for the providers. It is an accepted norm that for the maintenance of clinical skills and competencies, primary care providers require opportunity for acquired knowledge to be reinforced through refresher training activities [22]. Our experience in this project was similar as the evaluation scores after 9 months illustrated that while the skills and knowledge base of the primary care personnel who had been trained remained higher than baseline, it was nevertheless significantly less than the immediate post-training scores. Therefore, a structured programme of refresher workshop is a recommended procedure for dealing with this inevitable decay in skills and knowledge. Furthermore, we recognize that the knowledge and skills acquired during training may not necessarily translate into an actual change in clinical practice by trained providers. Thus, the scheduled supervision and monitoring activities conducted during this project, which included direct observation of clinical encounters and debriefing sessions with the providers, was designed to provide feedback on clinical skills and reinforce fidelity to the specifications of the mhGAP-IG.

A potential barrier to the implementation of any mental health service in Nigeria is the widespread stigmatization of mental disorders and of persons suffering from these disorders. Stigma may create a barrier to service use so that even when a service is available, persons in need may shy away from its use as a way of avoiding the associated stigma. The implementation of this project took cognizance of this observation and attempted to minimize its impact through public engagement activities conducted in three specific ways: 1) mental health enlightenment talks conducted by providers to attendees while waiting to be seen at the primary care clinics; 2) through meetings with community opinion leaders; and 3) the distribution of public enlightenment posters and flyers in schools, clinics and other selected public places. The dramatic increase in the number of persons seeking care for mental health conditions, some for the first time in a long time since onset of the condition, suggests that these engagement activities did improve mental health service uptake possibly through the reduction of perceived stigma. Even though we did not measure it, we are of the formed opinion that, due to increased contact, stigma of mental illness also reduced among the service providers, an important step in having a sustainable scaling up effort. The implementation of this project thus benefited from our knowledge of previous observations suggesting that failure to mobilize and adequately engage the community could be a potential barrier to the successful integration of mental health service into primary care [9].

We observed that relatively small numbers of clients were seen for MNS conditions despite the training of the providers to deliver improved service and the public engagement activities designed to inform the public about the availability of service. This was also in spite of the fact that the clinics were serving communities with relatively large population. This finding most likely reflects the observation previously made by others that even with continuous and sustained community engagement, ensuring acceptability and an increase in service utilization is often a long and slow process, whereby the numbers are initially modest and then slowly build up over time [23].

This pilot project utilized as much as was reasonably possible, the existing structure in place in the State. For example, the existing PHC co-ordinators who are usually non-specialist physicians or in the absence of a medical doctor, the most senior nurse, were recruited, trained and utilized as facilitators for the cascade training. They also served as supervisors, providing support and supervision across the clinics in their respective LGAs, as well as serving as links between the primary care staff and the mental health professionals from the tertiary facilities to facilitate referrals and seek guidance with respect to difficult cases.

A common refrain in the discourse about the challenges of integrating mental health into general medical services is the insufficient funding available to effectively implement integration. The utilization of a cascade training model using mid-level trainers who are trained by available mental health specialists could provide a cost effective and viable template for rolling out this model on a larger scale across the country, and indeed, in other LMIC. Such specialists could come from within the implementing country, when they are available, or from neighboring countries. They are required for a relatively short period of time to train a pool of local trainers from available health personnel who may be general physicians and senior nurses. The trained trainers thus become a valuable resource to train frontline providers as well as offer support and supervision.

An important observation is the increase in policy attention that resulted from the activities conducted in the process of implementing the demonstration project. In particular, the high profile policy attention by the nation’s Council of Health was not anticipated at the start of programme implementation. It does however show that in trying to advocate for improved policy attention to mental health service, particularly in LMIC, opportunities to demonstrate community need and the impact that improved service delivery can make to the lives of affected persons should be sought and utilized effectively. Apparently, policy makers need to be convinced about the reality of unmet needs and the fact that simple and affordable interventions are available.

Some concerns have been raised about the risk of focusing on a narrow biomedical approach to the integration of mental health into primary care. In this regard, seven elements have been highlighted as critical to ensuring that misplaced emphasis on a narrow biomedical approach is not adopted in efforts to carry out integration [13]. Most of these possible pitfalls were avoided in the design of this project. Firstly, the mhGAP-IG that was used for training and subsequently to aid routine clinical practice in primary care, places equal emphasis on both psychotherapeutic and pharmacological interventions. This was emphasized during the training workshops during which the utilization of available community resources to provide psychosocial support and the delivery of effective rehabilitation were also emphasized. Secondly, both the supervisory and referral networks deployed here were structured in a form of stepped care approach: from the primary care workers, to the PHC co-ordinators and finally to the mental health professionals at the tertiary facilities. Thirdly, the supervisory approach was conceived as ‘supportive supervision’ which provided continuous hands-on training, support and guidance to the primary care workers. Lastly, there was robust community engagement before and during the project while efforts were also made to influence relevant policy changes to facilitate an enabling environment for the successful implementation and the future scaling up of mental health service using this model.

## Conclusions

This project has demonstrated the feasibility of scaling up mental health services in primary care settings in Nigeria, using the mhGAP-IG and a cascade-training model. This format of training for PHC workers ensures maximal coverage, especially in low and middle income settings where there are few specialists.

## Additional file

Additional file 1:
**Appendix 1.**
