# Supplementary material for: Integrating mental health into primary care in Nigeria: report of a demonstration project using the mental health gap action programme intervention guide
Source: BMC Health Serv Res. 2015 Jun 21;15:242. doi: 10.1186/s12913-015-0911-3 (PMC4475323; doi:10.1186/s12913-015-0911-3)
Supplement: Additional file 1: — Appendix 1. [file 12913_2015_911_MOESM1_ESM.doc]

#
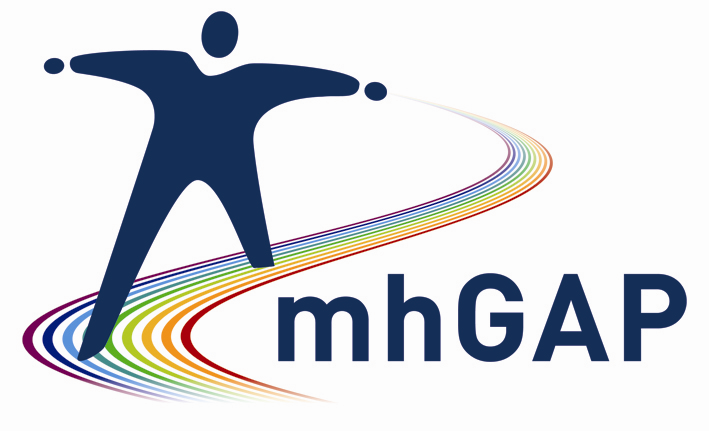
**Pre - Test for PHC workers Training on the mhGAP**

Duration of the test: 45 min.

Date of the test:

Please write initials here ____________________________________________________

Please write your job qualification here _________________________________________

| A. Put **✓** in the correct column. | True | False |
| --- | --- | --- |
| 1. People with mental disorders usually cannot make decisions concerning their health |  |  |
| 1. People with mental disorders are best cared for in mental hospitals |  |  |
| 1. Everyone with depression should be treated by antidepressants |  |  |
| 1. Dementia is a normal part of ageing |  |  |
| 1. Providing brief advice to people who have alcohol problems is effective |  |  |
| 1. Mental disorders are common in children and adolescents |  |  |
| 1. To stop acute seizures, diazepam by intramuscular route is the routine treatment of choice |  |  |
| 1. Severe chronic depression in a mother may lead to developmental delay in her children |  |  |
| 1. If the child shows over-activity and inattention, then medication is usually needed |  |  |
| 1. Vitamin injections should be routinely used for somatic complaints with no organic cause |  |  |
| 1. Asking people about suicidal thoughts increases the likelihood of suicide |  |  |

B. Please mark **✓** for the correct answer. There is only one correct answer for each question.

1. Which one of the following statements concerning depression is correct
   1. Depression often presents with vague physical pain and fatigue
   2. Depression often presents with delusions and hallucinations
   3. Depression often presents with confusion
2. Concerning antidepressants which of the following is correct
3. The treatment should be continued even if the person suddenly becomes manic
4. The treatment should be continued for 2-3 months
5. The treatment should usually only be offered if the depression affects the person’s daily functioning
6. Which of the following messages should be given to a person with depression
7. Try to reduce your physical activity as much as possible
8. Try to participate in social activities as much as possible
9. Try to sleep as much as possible
10. A 22 years old girl says that she hears voices that no one else can hear and is convinced that someone wants to hurt her, which of the following disorders is most likely present
11. Psychosis
12. Depression
13. Mania
14. Concerning the management of acute psychosis
15. Medicines by injection will be required for most cases
16. The person needs to be followed up at frequent interval
17. The person should always be restrained (e.g. chained)
18. After a suicide attempt
19. Leave the person alone resting in a quiet room
20. Restrain visits from family and friends
21. Remove means of self-harm
22. Which of the following statements is correct concerning alcohol use
23. If people drink alcohol every day of the week, they are alcohol dependent
24. Alcohol use cannot cause seizures
25. People can have an alcohol problem even if they only drink once in one month
26. Concerning drug use disorder which of the following is correct
27. Imprisonment is the most effective intervention
28. Mothers who use drugs should not breastfeed
29. Discussing with the person their ideas about perceived benefits and potential harms of drug use is useful
30. Which of the following statements concerning pharmacological treatment for people with mental disorder is correct
31. You usually do not need to obtain consent since the person does not understand
32. Antidepressants should only be given to adolescents after trying psychosocial treatment
33. Once the antipsychotic treatment starts, the person needs to continue taking the drug throughout life

D. Please put **✓**  in front of your choice:

| Subject | Not true | Somewhat true | Very true |
| --- | --- | --- | --- |
| 21. I feel worried about working with people with mental illness |  |  |  |
| 22. I like to interact with people with mental illness |  |  |  |
| 23. I feel competent to identify and treat people with  Mental illness in my clinic |  |  |  |

Thank you for your kind assistance
